# Fingolimod Increases CD39-Expressing Regulatory T Cells in Multiple Sclerosis Patients

**DOI:** 10.1371/journal.pone.0113025

**Published:** 2014-11-20

**Authors:** Nathalie Muls, Hong Anh Dang, Christian J. M. Sindic, Vincent van Pesch

**Affiliations:** 1 Neurochemistry Unit, Institute of Neuroscience, Université catholique de Louvain, Brussels, Belgium; 2 Cliniques Universitaires Saint-Luc, Brussels, Belgium; Klinikum rechts der Isar der Technischen Universitaet Muenchen, Germany

## Abstract

**Background:**

Multiple sclerosis (MS) likely results from an imbalance between regulatory and inflammatory immune processes. CD39 is an ectoenzyme that cleaves ATP to AMP and has been suggested as a novel regulatory T cells (Treg) marker. As ATP has numerous proinflammatory effects, its degradation by CD39 has anti-inflammatory influence. The purpose of this study was to explore regulatory and inflammatory mechanisms activated in fingolimod treated MS patients.

**Methods and Findings:**

Peripheral blood mononuclear cells (PBMCs) were isolated from relapsing-remitting MS patients before starting fingolimod and three months after therapy start. mRNA expression was assessed in *ex vivo* PBMCs. The proportions of CD8, B cells, CD4 and CD39-expressing cells were analysed by flow cytometry. Treg proportion was quantified by flow cytometry and methylation-specific qPCR. Fingolimod treatment increased mRNA levels of CD39, AHR and CYP1B1 but decreased mRNA expression of IL-17, IL-22 and FOXP3 mRNA in PBMCs. B cells, CD4^+^ cells and Treg proportions were significantly reduced by this treatment, but remaining CD4^+^ T cells were enriched in FOXP3^+^ cells and in CD39-expressing Tregs.

**Conclusions:**

In addition to the decrease in circulating CD4^+^ T cells and CD19^+^ B cells, our findings highlight additional immunoregulatory mechanisms induced by fingolimod.

## Introduction

Multiple sclerosis (MS) is a chronic inflammatory disease of the central nervous system (CNS) characterized by demyelination and neurodegeneration. A key event in MS pathogenesis is the peripheral activation of autoreactive lymphocytes. These cells damage the blood-brain barrier (BBB), enter the CNS and cause local inflammation.

Disease-modifying treatments are designed to slow down disease progression by reducing the relapse rate and the accumulation of new lesions on MRI. Fingolimod (Gilenya, Novartis), a sphingosine analogue, was the first once-daily oral drug approved for the treatment of relapsing-remitting MS (RRMS). Both sphingosine and fingolimod are phosphorylated to their active forms by intracellular sphingosine kinases. Phosphorylated fingolimod (fingolimod-P) mimics the activity of sphingosine-1-phosphate (S1P) and acts as an agonist on 4 out of its 5 receptors [1]. Thanks to their S1P receptors, acting as sensors, lymphocytes follow the S1P gradient to exit secondary lymphoid organs and reach the blood flow. Prolonged binding of fingolimod-P to its receptors induces their internalization and degradation [2]. Fingolimod therefore reversibly retains most lymphocytes subsets within the lymph nodes. In MS patients, fingolimod reduces the proportion of pro-inflammatory T helper (Th) cells producing interleukin-17 (IL-17), in the circulating blood [3]. The effects of fingolimod on T regulatory cells (Tregs) are still incompletely described. Animal studies have shown that fingolimod induces the conversion of CD4^+^FOXP3^-^ cells into CD4^+^FOXP3^+^ cells [4], [5]. In contrast, one report supports that fingolimod decreases the immunosuppressive activity of Tregs in the context of graft-versus-host disease [6].

CD4^+^CD25^hi^FOXP3^+^ regulatory T cells are a subset of cells specialized in the suppression of activation and proliferation of effector T cells. Therefore, this subset is of particular importance in limiting autoimmunity. Tregs are characterized by the expression of the cellular marker CD25 and the transcription factor FOXP3. However, in humans, these two markers are not specific for Tregs as they are also expressed on effector T cells after stimulation. Therefore, it remains uneasy to distinguish natural Tregs and recently activated T cells. Analysing the methylation status of the *FOXP3* locus might be of particular interest to quantify these cells. Indeed, the first intron of *FOXP3* (*FOXP3i1*) shows complete demethylation in natural Tregs (nTregs) [7]. CD39 is an immunoregulatory molecule expressed by effector/memory-like Tregs (T_REM_) that decreases the extracellular level of ATP, converting it into AMP [8]. Under certain conditions, its expression can be regulated by the transcription factor Aryl Hydrocarbon Receptor (AHR) [9]. CD39-expressing Tregs are of particular interest in MS as they have been shown to specifically inhibit Th17 cells [10].

The aim of our study was to investigate the effects of fingolimod on pro- and anti-inflammatory Th17- and Treg-related immune markers, by analysing their *ex vivo* expression profile in treated MS patients. We then analysed the effects of fingolimod therapy on the proportions of various cell subsets, notably of CD39-expressing cells.

## Materials and Methods

### Subjects and sample collection

Blood samples were obtained from 16 patients with RRMS before starting fingolimod therapy, and after 3 months of treatment (0.5 mg daily). The study was approved by the local ethics committee and written informed consent was obtained from all patients. Peripheral blood mononuclear cells (PBMCs) were also collected from ten age- and sex-matched healthy controls (HC). Basic demographic features are summarized in Table S1. All patients displayed the expected lymphocyte count reduction three months after starting fingolimod treatment.

### Methylation Specific-qPCR (MethylS-qPCR) for FOXP3i1

Genomic DNA (gDNA) was prepared from frozen pellets containing 10^6^ total PBMCs with the PureLink DNA Mini Kit (Invitrogen). One µg of gDNA was treated with sodium bisulfite using the EpiTect Plus DNA Bisulfite Kit (Qiagen). Real-Time PCR amplification of methylated and demethylated *FOXP3i1* sequences was performed in a final volume of 25 µl with the Rotor-Gene Probe PCR Kit (Qiagen), 300 nM of each primer and 100 mM of probe in a 72-well rotor on Rotor-Gene PCR 6000 Realtime Analyser (Corbett Life Science). Two-step thermal cycling was started with a first denaturation at 95°C for 3 minutes followed by 45 cycles at 95°C for 3 seconds and 64°C for 30 seconds. Sequences of primers and probes are indicated in Table S2. The percentage of demethylated sequences is calculated as follows: 2∧(Ct methylated −Ct demethylated)/[2∧(Ct methylated −Ct demethylated)+1]*100.

### Fluorescence activated cell sorting (FACS)

Thawed PBMCs were resuspended in PBS with 1% foetal calf serum and 2 mM EDTA. Cells were stained for surface antigens with anti-human CD4 (eBioscience), CD8, CD19, CD39 and CD25 (BioLegend) antibodies, then fixed and permeabilized overnight prior to FOXP3 staining (clone 236A/E7, eBioscience). Lymphocytes were gated according to their FSC/SSC (forward/side scatter) profile. Median fluorescent intensity (MFI) was analysed on the FOXP3 channel for CD4^+^ cells and on the CD39 channel for CD4^+^CD25^hi^FOXP3^+^ cells. Acquisition was performed on a BD LSR Fortessa instrument (BD Biosciences). All data were analysed using FlowJo software (Tree Star Inc.). Conditions were assayed in duplicate.

### cDNA analysis and qPCR experiments

RNA from PBMCs was isolated using the miRNeasy mini kit (Qiagen) according to the manufacturer's protocol and reverse transcribed to cDNA using Superscript Reverse Transcriptase (Invitrogen). Quantitative PCR (qPCR) assays were performed using Absolute qPCR SYBR Green mix (Thermo Scientific) on a Rotor-Gene 6000 (Corbett Life Science). The relative amount of transcripts was determined by normalizing for Abelson gene (ABL) using the comparative Ct method (2^−ΔΔCt^) [11]. Results are expressed relative to the mean of the healthy control patients (HC) set at 1. Sequences of primers are detailed in Table S3.

### Statistical analysis

Statistical analysis was performed using the GraphPad Prism 5 software. To test for differences before and after ivMP treatment, a non-parametric Wilcoxon's signed rank test was performed. MS patients and HC were compared using the non-parametric Mann Whitney test. P-values ≤0.05 were considered as statistically significant.

## Results

### Ex vivo mRNA expressions of IL-17, IL-22, CD39, FOXP3, AHR and CYP1B are affected by fingolimod in PBMC

**Figure 1:**
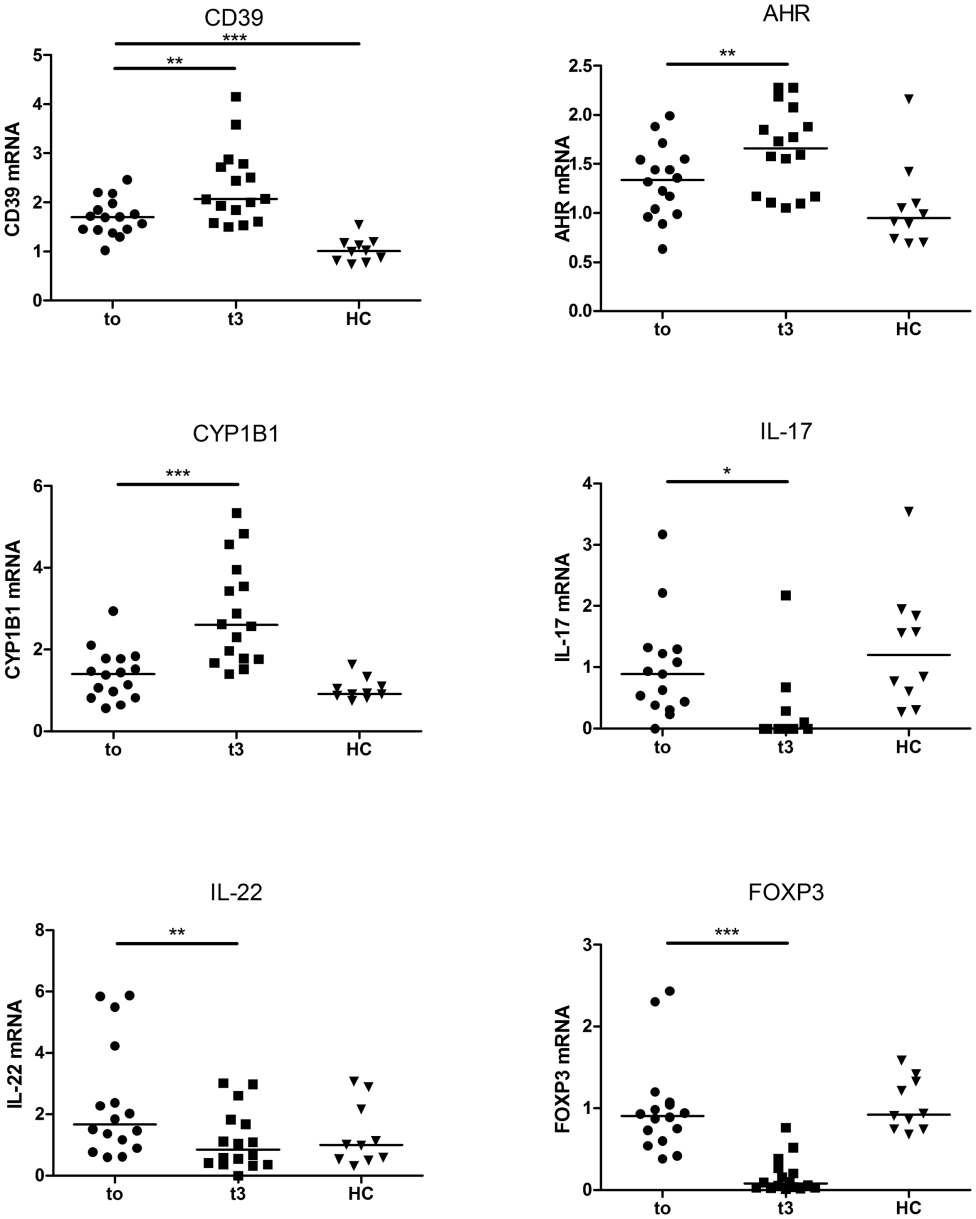
*Ex vivo* mRNA expression in total PBMCs from fingolimod-treated patients. Scatter dot plots show relative mRNA expression levels in PBMCs from MS patients before (t0) or after 3 months (t3) of treatment by fingolimod, and healthy controls (HC) analysed by q-PCR. For each target, individual mRNA levels were expressed as relative values to the mean level of the control group. The mRNA expression levels of CD39 (p = 0.0033), as well as of AHR (p = 0.007) and CYP1B1, an AHR-induced gene (p<0.0001) were increased after fingolimod treatment. On the contrary, fingolimod reduced the mRNA levels of IL-17, IL-22 and FOXP3. Horizontal lines represent the median value in all subgroups. *, ** and *** indicate p-values of ≤0.05, ≤0.01 and ≤0.001 respectively.

In comparison to HC, the only significant difference was an increase of CD39 mRNA level in MS patients (Figure 1). Indeed, median mRNA level was 70% higher in MS patients (p = 0.0002). The mRNA expression levels of CD39 (p = 0.0033), as well as of AHR (p = 0.007) and CYP1B1, an AHR-induced gene (p<0.0001) were increased after fingolimod treatment. On the contrary, fingolimod reduced the mRNA levels of IL-17, IL-22 and FOXP3. The most dramatic decrease was observed for IL-17 mRNA, which was undetectable after therapy in most patients (n = 11/16) (p = 0.024).

### CD4^+^ T and B cell proportions are reduced in circulating lymphoid cells after fingolimod treatment

**Figure 2:**
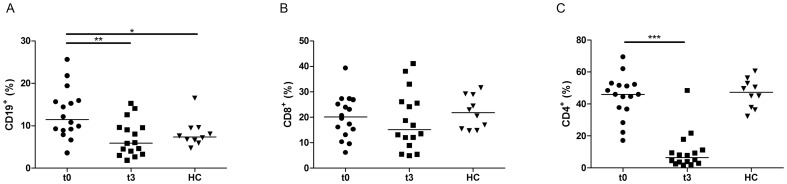
Circulating T and B cell proportions in patients treated by fingolimod. PBMCs of MS patients before (t0, n = 16) or after three months of treatment by fingolimod (t3, n = 16) and healthy controls (HC, n = 10) were analysed *ex vivo* by flow cytometry. The percentage of (A) CD19^+^ B cells, (B) CD8^+^ and (C) CD4^+^ T cells within lymphoid cells is shown. The proportion of B cells and CD4^+^ T cells decreased after fingolimod while the proportion of CD8^+^ T cells was not significantly modified. The horizontal lines represent the median value in all subgroups. *, ** and *** indicate p-values of ≤0.05, ≤0.01 and ≤0.001 respectively.

As fingolimod inhibits the egress of lymphocytes from lymph nodes, we wanted to further quantify cellular subsets present in PBMCs. The proportion of B cells, characterised by the CD19 surface marker, was reduced by 50% with fingolimod treatment (p = 0.0012). Before therapy, CD19^+^ B cells represented 11.45% of the circulating lymphoid cells. This proportion was reduced to 5.9% under treatment (Figure 2, Table 1). In contrast, the proportion of CD8^+^ T cells did not show a significant decrease.

**Table 1:** Immune cell subpopulations in MS patients before (t0) or after three months of fingolimod treatment (t3) and healthy controls analysed by *ex vivo* flow cytometry.

	t0	t3	p-value (t0 vs t3)	HC	p-value (t0 vs HC)
CD4 (%of lymphoid cells)	46.03 (17.2–69.6)	6.4 (1.7–48.5)	**0.0006**	47.3 (32.3–60.6)	0.770
CD8 (%of lymphoid cells)	20.1 (6.2–39.4)	15.18 (4.9–41.1)	0.4851	21.73 (14.7–31.5)	0.544
CD19 (%of lymphoid cells)	11.45 (3.6–25.7)	5.9 (1.8–15.25)	**0.0012**	7.36 (4.7–16.5)	**0.025**
FOXP3^+^ (%of CD4^+^)	7.92 (3.46–11.2)	11.78 (1.35–20.6)	**0.0411**	5.45 (2.93–10.6)	0.087
CD4^+^CD25^hi^FOXP3^+^ (%of lymphoid cells)	3.2 (1.67–8.26)	0.68 (0.06–4.34)	**0.0029**	2.63 (1.98–5.01)	0.580
CD25^hi^FOXP3^+^ (%of CD4^+^)	8.33 (3.73–11.9)	11.45 (1.17–19.75)	0.0929	6.27 (4.4–10.95)	0.087
CD39^+^ (%of CD4^+^CD25^hi^FOXP3^+^)	43.4 (17.9–77.3)	76.2 (29.9–93.6)	**0.0005**	26.9 (10.1–53.15)	**0.0425**

The median and range are shown. Lymphoid cells were gated according to their FSC/SSC profile.

**Figure 3:**
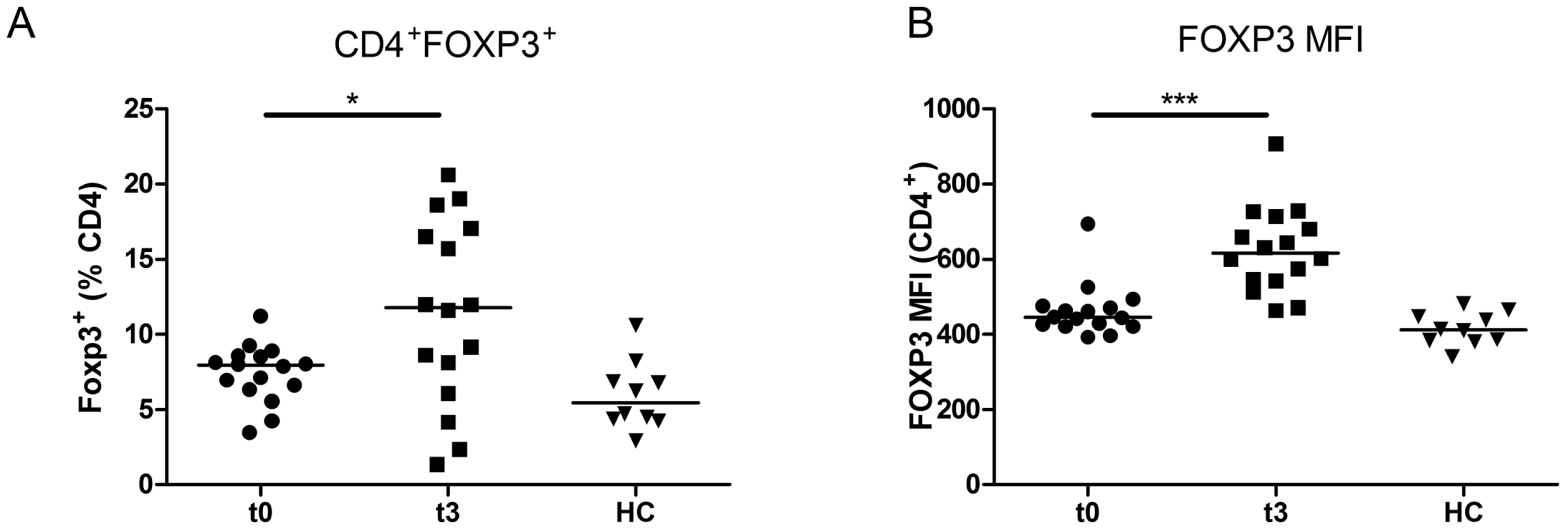
FOXP3 expression by CD4^+^ T cells of fingolimod-treated MS patients before (t0, n = 16) or after three months of treatment by fingolimod (t3, n = 16) and healthy controls (HC, n = 10) analysed *ex vivo* by flow cytometry. (A) The percentage of FOXP3-expressing CD4^+^ cells was increased in 12 out of 16 patients and associated with an increase (B) of FOXP3 MFI values. * and *** indicate p-values of ≤0.05 and ≤0.001 respectively.

Fingolimod decreased the proportion of CD4^+^ T cells by 7-fold (p = 0.0006) (Figure 2). Before treatment, CD4^+^ T cells accounted for 46% of the circulating lymphoid cells, but only for 6.5% after three months of fingolimod therapy. In contrast, the proportion of FOXP3-expressing CD4^+^ cells was increased in 12 out of 16 patients (p = 0.04, Figure 3A). This was associated with a median increase of 40% in the FOXP3 MFI values (p = 0.0005, Fig. 3B).

### Regulatory T cells proportion is decreased in PBMCs after fingolimod treatment

**Figure 4:**
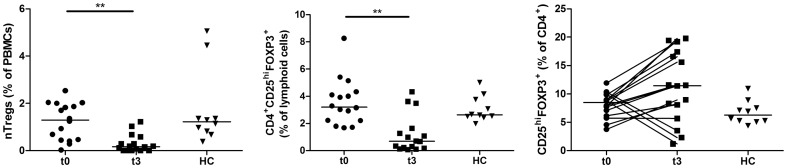
Regulatory T cells in PBMCs of fingolimod-treated patients. Regulatory T cells of MS patients before (t0, n = 16) or after three months of treatment by fingolimod (t3, n = 16) and healthy controls (HC, n = 10) were analysed *ex vivo* by MethylS-qPCR (A) to quantify nTregs with demethylated *FOXP3i1*. The proportion of circulating CD4^+^CD25^hi^FOXP3^+^ Tregs was also measured by flow cytometry and expressed as a percentage of lymphoid cells (B). The proportion of CD25^hi^FOXP3^+^ cells was also expressed as a percentage of CD4^+^ T cells (C). nTreg proportion among total PBMCs was decreased by the treatment, as well as the proportion of Tregs within lymphoid cells. CD25^hi^FOXP3^+^ Tregs were however enriched within the CD4^+^ cell population in 12 out of 16 patients. The horizontal lines represent the median value in all subgroups. ** indicates a p-value of ≤0.01.

As regulatory T cells play a central role in maintaining self-tolerance, we then analysed the Treg proportion within total PBMCs or CD4^+^ T cells. Natural Tregs were quantified by analysing the epigenetic status of the first intron of *FOXP3* using methylation specific PCR (Figure 4A). CD4^+^CD25^hi^FOXP3^+^ Treg phenotype was also examined by flow cytometry (Figure 4B). Using both MethylS-qPCR (p = 0.041) and flow cytometry (p = 0.0029), we showed that the proportion of Tregs among total PBMCs was decreased by the treatment. However, the proportion of CD25^hi^Foxp3^+^ Tregs within the CD4^+^ cell population increased in 12 patients out of 16 (Figure 4C, Table 1). Only 3 patients displayed a reduction in their Treg proportion while on treatment by fingolimod and one patient did not show change in this parameter.

### CD39^+^ Tregs increase after fingolimod

**Figure 5:**
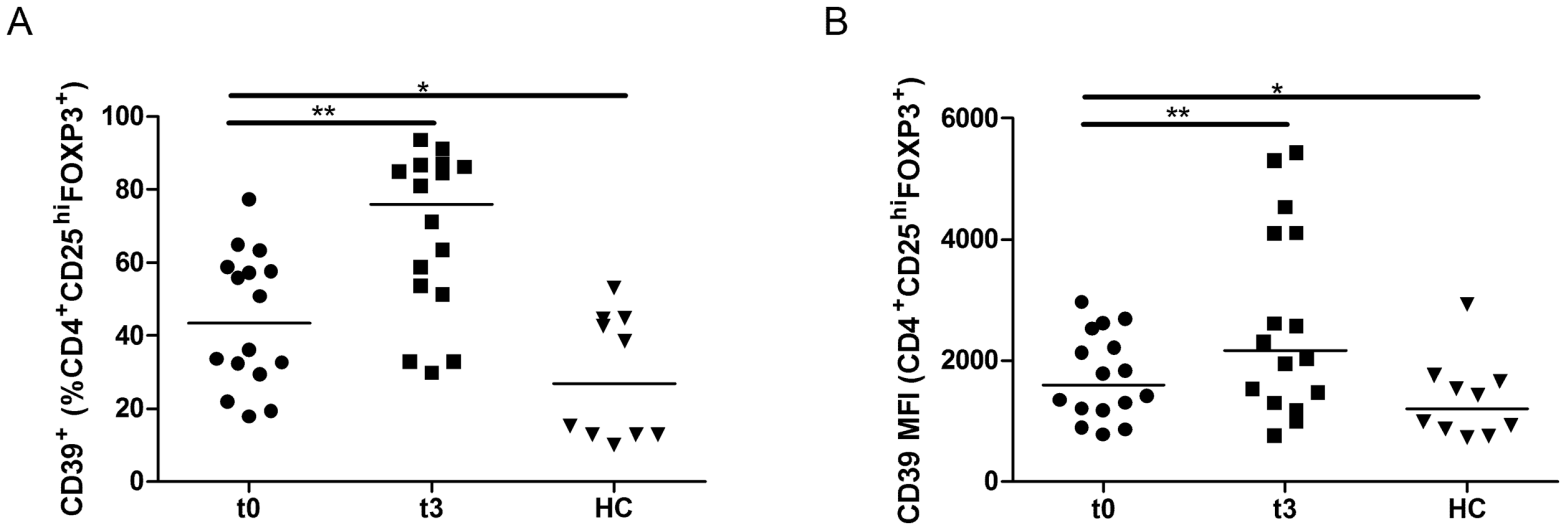
Proportion of CD39-expressing cells within CD4^+^CD25^hi^FOXP3^+^ T cells of fingolimod-treated patients. (A) The percentage of CD39 expression within the population CD4^+^CD25^hi^FOXP3^+^ T cells of MS patients before (t0, n = 16) or after three months of treatment by fingolimod (t3, n = 16) and healthy controls (HC, n = 10) was analysed *ex vivo* by flow cytometry. The proportion of CD39-expressing Tregs was increased after three months of fingolimod treatment and was associated with (B) an increase in the MFI of CD39. The horizontal lines represent the median value in all subgroups. * and ** indicate p-values of ≤0.05 and ≤0.01 respectively.

CD39^+^ Tregs have been shown to specifically inhibit IL-17 producing cells. Since Th17 cells exert pathogenic effect in MS, we quantified CD39^+^ Tregs after treatment by fingolimod. The proportion of CD39-expressing CD4^+^CD25^hi^FOXP3^+^ Tregs was increased in MS patients in comparison to HC (43.4% *versus* 26.9%; p = 0.009). After three months of fingolimod treatment, the proportion of CD39^+^ Tregs further increased from 43.4% to 76.2%, representing a median increase of 77%. This was associated with an increase in the MFI of CD39 (Figure 5A and 5B and Table 1). The proportion of CD4^+^FOXP3^-^CD39^+^ cells was affected to a lesser extent after fingolimod treatment rising from 5.1% to 7.2% (median increase of 41%). On the contrary, within the CD8^+^ and CD19^+^ populations, the proportion of CD39^+^ cells and the CD39 MFI were not increased by the treatment (Figure S1).

## Discussion

MS is a demyelinating disease of the CNS thought to be mediated in part by autoreactive lymphocytes. The role of proinflammatory responses in MS has been largely investigated and the implication of the Th17 lineage has been demonstrated in animal and human studies [12]–[14]. Fingolimod has been approved as a disease-modifying drug in the treatment of RRMS. Fingolimod may participate to various immunoregulatory processes other than lymphocytes retention as the S1P_1_ signalling is involved in many other physiological responses [15].

Here, we show that fingolimod significantly reduces the mRNA level of IL-17 in total PBMCs. This result is in agreement with the finding that fingolimod decreases the amount of circulating IL-17 producing cells [3]. A similar effect is shown for IL-22. This IL-10 cytokine family member plays both protective and pathogenic roles in autoimmune diseases but its involvement in MS is still matter of debate. [16]–[18] IL-22 is not crucial in the development of the murine experimental model of MS [19]. However, *IL22RA2* is a susceptibility gene for MS [20], [21].

Interestingly, mRNA levels of CD39, AHR and CYP1B1, an AHR-induced gene are increased in fingolimod-treated patients. CD39 is an ectonucleotidase that hydrolyses ATP to AMP. Since ATP acts as an inducer of proinflammatory cytokines, its cleavage has anti-inflammatory consequences [22]. CD39 has been described as a novel functional marker of regulatory effector/memory-like T (T_REM_) cells [8]. AHR is a member of the family of basic helix-loop-helix transcription factors. Upon binding of exogenous agents, AHR mediates transcriptional responses of a wide variety of genes, named AHR gene battery, which includes CYP1B1 [23]. AHR has also been demonstrated to influence the expression of IL-22 and CD39 [9], [24]. In this study, we show an increase in the mRNA expression levels of CD39 and CYP1B1. Thus, AHR activation might be an additional anti-inflammatory mechanism induced by fingolimod. AHR activation was shown to exacerbate or to suppress EAE depending on its ligand [24], [25]. EAE suppression by AHR was associated with expansion of Treg cells. Therefore, the effects of fingolimod on AHR might have functional relevance, in addition to its effects on the redistribution of circulating immune cells.

We have also shown that the *ex vivo* levels of FOXP3 mRNA in PBMCs are drastically reduced in contrast to the increase in the proportion of FOXP3^+^ cells within CD4^+^ T cells in fingolimod-treated patients. FOXP3 is the transcription factor of Tregs but is also transiently expressed upon activation of effector T cells. Therefore, its decreased mRNA level is likely due to the large reduction of CD4^+^ cell count observed by flow cytometry. Indeed, in agreement with previous studies, CD4^+^ and B cells proportions are decreased by fingolimod while CD8^+^ T cells are less affected [26], [27]. This is consistent with the observation that T_EM_ cells, which are mainly CD8^+^ cells, are not retained in lymph nodes in the presence of fingolimod, in contrast to T_CM_ cells, which are predominantly CD4^+^ cells [28].

Divergent findings have been published regarding the effects of fingolimod on the proportion of Tregs [29], [30]. Due to the lack of specific cell surface Treg markers in humans, we used *FOXP3i1* demethylation to assess the effect of fingolimod on circulating natural Tregs. By flow cytometry, we confirm that the proportion of Tregs within lymphoid cells is reduced by fingolimod. However, the proportion of CD25^hi^Foxp3^+^ cells within the CD4^+^ subpopulation increases in 12 treated patients out of 16. It has been suggested that fingolimod induces FOXP3 expression in CD4^+^FOXP3^-^ T cells and causes an increase in FOXP3^+^ Treg cells [4], [5]. Here, we show an increase in the proportion of FOXP3^+^ cells within the CD4^+^ cell population, as well as an increase in median FOXP3 expression level, as indicated by higher MFI values.

Beneficial effects of fingolimod might be additionally mediated by modulating the function of Tregs. In order to investigate this, we analysed the expression of CD39 by Tregs. The proportion of CD39^+^ Tregs is significantly increased by fingolimod. Surface expression of CD39 in Tregs is also increased by the treatment. It has been suggested that CD39^+^ Tregs specifically inhibit Th17 cells. Therefore, their increased number might be an additional mechanism by which fingolimod attenuates inflammation and mediates its therapeutic benefits.

In summary, we have shown that CD39, AHR and CYP1B1 mRNA levels are increased in PBMCs following fingolimod treatment while IL-17 mRNA is decreased. Fingolimod induces a large decrease of B and CD4^+^ cells but remaining CD4^+^ T cells are enriched in FOXP3^+^ cells. Furthermore, the proportion of CD39-expressing Tregs within the CD4^+^ subpopulation is increased by the treatment, thereby possibly providing an additional therapeutic benefit of this drug. Indeed, apart from its effects on lymphocyte egress from lymph nodes, experimental data points towards an effect of fingolimod on Treg development. Signalling through the S1P1 receptor has been shown to inhibit Foxp3^+^ Treg differentiation, by down-regulating SMAD3 activity [31]. This consequently counteracts TGF-β receptor-mediated signalling, which is required for Treg differentiation. Inhibition of S1P1 signalling by fingolimod might therefore regulate the balance towards Treg development, as observed in our study. It has also been shown that treatment with fingolimod induces TGF-β in splenocyte cell cultures, which could also be a mechanism involved in the increased proportion of CD39-expressing Tregs in fingolimod-treated patients [32]. Finally, our data showing an induction of CYP1B1 mRNA following treatment by fingolimod suggests that AHR, another transcription factor involved in Treg differentiation and CD39 expression is activated, providing an alternative hypothesis for our observations [9], [25]. Studies are ongoing regarding the mechanisms by which fingolimod induces CD39-expressing cells.

## Supporting Information

Figure S1 CD39-expressing cells within other cell subpopulations. CD39 expression was analysed by flow cytometry in CD4^+^FOXP3^-^ T cells (A), CD19^+^ B cells (B) and CD8^+^ T cells (C). The proportion of CD4^+^FOXP3^-^CD39^+^ cells increases slightly following treatment by fingolimod. The treatment did not increase the proportion of CD39^+^ cells within the CD8^+^ and CD19^+^ populations. The horizontal lines represent the median value in all subgroups. * and ** indicate p-values of ≤0.05 and ≤0.01 respectively.
(TIF)

Table S1 Main demographic features of patient and healthy control groups.
(DOCX)

Table S2 Primers and probes sequences for MS-qPCR amplification.
(DOCX)

Table S3 Primer sequences for qPCR amplification.
(DOCX)
