# Supplementary material for: Fingolimod Increases CD39-Expressing Regulatory T Cells in Multiple Sclerosis Patients
Source: PLoS One. 2014 Nov 20;9(11):e113025. doi: 10.1371/journal.pone.0113025 (PMC4239031; doi:10.1371/journal.pone.0113025)
Supplement: Table S1 — Main demographic features of patient and healthy control groups. (DOCX) [file pone.0113025.s002.docx]

|  | RRMS Patients | Healthy Controls |
| --- | --- | --- |
| Number | 16 | 10 |
| Mean age± S.D. | 40,6±10,7 | 36,5±10,9 |
| % Female | 56 | 60 |

**Supplementary Table 1:** Main demographic features of patient and healthy control groups.
