# Supplementary material for: Fingolimod Increases CD39-Expressing Regulatory T Cells in Multiple Sclerosis Patients
Source: PLoS One. 2014 Nov 20;9(11):e113025. doi: 10.1371/journal.pone.0113025 (PMC4239031; doi:10.1371/journal.pone.0113025)
Supplement: Table S2 — Primers and probes sequences for MS-qPCR amplification. (DOCX) [file pone.0113025.s003.docx]

| Form detected | Primer type | Sequence (5’ to 3’) |
| --- | --- | --- |
|  | sense | CTCTTCTCTTCCTCCGTAATATCG |
| Methylated FOXP3 intron 1 | Anti-sense | GTTATTGACGTTATGGCGGTC |
|  | FAM-Eclipse Probe * | AAACCCGACGCATCCGAC |
|  | sense | TCTACCCTCTTCTCTTCCTCCA |
| Demethylated FOXP3 intron 1 | Anti-sense | GATTTTTTTGTTATTGATGTTATGGT |
|  | FAM-Eclipse Probe * | AAACCCAACACATCCAACCA |

- Underlined nucleotides correspond to LNA modified bases (Eurogentec).

**Supplemental Table 2:** Primers and probes sequences for MS-qPCR amplification.
