# Supplementary material for: Fingolimod Increases CD39-Expressing Regulatory T Cells in Multiple Sclerosis Patients
Source: PLoS One. 2014 Nov 20;9(11):e113025. doi: 10.1371/journal.pone.0113025 (PMC4239031; doi:10.1371/journal.pone.0113025)
Supplement: Table S3 — Primer sequences for qPCR amplification. (DOCX) [file pone.0113025.s004.docx]

| Target mRNA | Sense | Nucleotide sequence |
| --- | --- | --- |
| ABL | Forward | 5' - AAA ACC TTC TCG CTG GAC CC - 3' |
|  | Reverse | 5' - TTT GGG CTT CAC ACC ATT CC - 3' |
| AHR | Forward | 5' - ACA ACC GAT GGA CTT GGG TC - 3' |
|  | Reverse | 5' - TGG CAG GAA AAG GGT TT - 3' |
| CD39 | Forward | 5' - GTG GAG TTC AAA ATA GAC ATC GTG - 3' |
|  | Reverse | 5' - CAG CAC CCA CAC CGC CTT CTC CCG CTT - 3' |
| CYP1B1 | Forward | 5' - AGA ACG TAC CGG CCA CTA TC - 3' |
|  | Reverse | 5' – GGC TGG TCA CCC ATA CAA G- 3' |
| FOXP3 | Forward | 5' - CAA CAT GGA CTA CTT CAA GTT C - 3' |
|  | Reverse | 5' - ACT TGT GCA GAC TCA GGT TGT - 3' |
| IL-17A | Forward | 5' - AAC AAC GAT GAC TCC TGG GAA - 3' |
|  | Reverse | 5' - GTT ATG GAT GTT CAG GTT GAC - 3' |
| IL-22 | Forward | 5' - AGG CTC AGC AAC AGG CTA AG - 3' |
|  | Reverse | 5' - TTT GCT CTG GTC AAA TGC AG - 3' |

**Supplementary Table 3:** Primer sequences for qPCR amplification.
